# Medical student syndrome: a bayesian reasoning failure

**DOI:** 10.1186/s12909-026-09018-9

**Published:** 2026-03-16

**Authors:** Ryan C. Clarkson, Naeem Paruk

**Affiliations:** 1 Forward Observations Applied Risk Division, Dublin, Ireland; 2 Department of Mathematical Sciences, Stellenbosch University , Stellenbosch, South Africa

**Keywords:** Medical Student Syndrome, Bayesian reasoning, Base rate neglect, Diagnostic bias, Medical education

## Abstract

**Background:**

Medical Student Syndrome (MSS) is often understood as a manifestation of illness anxiety disorder where medical students misinterpret benign symptoms as signs of a serious illness. Current explanations rely heavily on psychological factors; however, these fail to capture the systemic reasoning errors that underly MSS. This paper proposes a mixed model: that MSS results from a combination of the Bayesian reasoning failure of base rate neglect and psychological stressors.

**Methods:**

An observational cross-sectional study of medical students was conducted $\left(n=112\right)$. Participants completed an anonymous survey assessing self-reported MSS, responses to medical diagnostic scenarios modelled on Kahneman and Tversky’s “librarian or farmer” problem, and prioritisation of diagnostic reasoning components. Descriptive statistics and categorical analysis of qualitative responses were performed.

**Results:**

A history of MSS was reported by 56.3% of participants. Diagnostic accuracy was low for both medical scenarios (48.3% correct for differentiating between stroke and migraine; 33.3% for differentiating between pheochromocytoma and panic disorder) and the Bayesian reasoning task (41.1% correct). 71% of students who answered medical questions incorrectly also failed the Bayesian task, suggesting a shared underlying process. 14% of students with a history of MSS answered both questions correctly. Students ranked pathophysiology and symptoms of the presenting complaint as most important in diagnosis, and epidemiology as the least important (mean rank = 8.8/9). Self-analysis of errors showed that most students attributed their mistakes to poor probabilistic reasoning rather than a lack of knowledge.

**Conclusion:**

The findings support the explanation of MSS as partially a statistical reasoning error rather than a purely psychological condition. Training in Bayesian reasoning, integration of epidemiology into diagnostic teaching, and diversification of assessment formats may mitigate MSS and enhance diagnostic accuracy for medical students.

**Supplementary Information:**

The online version contains supplementary material available at 10.1186/s12909-026-09018-9.

## Introduction

Medical Student Syndrome (MSS) is commonly understood to be a form of Illness Anxiety Disorder (hypochondria), characterised by a health sciences student becoming concerned that they are showing signs of a serious or rare disease that they do not have. Whilst some research defines the student’s belief about their diagnosis as being connected to a pathology they have recently studied in class [1], this arbitrary limitation is not consistently applied across the literature and has not been applied to this research.

MSS is a common phenomenon with a wide-ranging prevalence reported in the literature (8.5% − 70%) [1, 2]. In Abdellatif et al. (2025) [3], 31% of the 300 students sampled admitted to experiencing it. This occurs despite popular knowledge of the phenomenon – in the same paper [3], over 60% of students knew of MSS. MSS increases anxiety, and in a cross-sectional study of 544 students, 39.7% reported that their grades were negatively impacted by MSS [1].

Physicians without formal training in epidemiology and biostatistics lack the skills necessary to interpret epidemiological data [4]. When academic physicians are singled out, a French study found that only 16.9% could calculate a likelihood ratio, and only 9.2% knew how to interpret and use it [5].

This appears to indicate a systemic issue in medicine that is not limited to just medical school. In diagnostic tasks, both students and physicians have been found to be prone to multiple types of bias, including base rate neglect [6]. This is supported by students’ own reflections on their medical training – a 2018 study found that many feel undereducated in statistics (120 students gave an average ranking of 2.68/4 for their ability to perform basic statistical analysis), and lack the confidence to apply statistical tools in research settings [7].

**Fig. 1:**
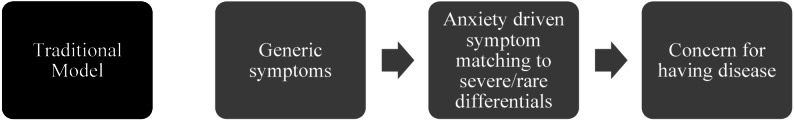
The traditional model for the process of MSS

Current explanations for MSS are insufficient and rely on correlation and a psychological explanation – that medical students who experience this are experiencing anxiety, burnout or are hypochondriacs [3, 8]. Figure 1 describes how vague symptoms turn into concerns for one’s health under this traditional framework.

This paper puts forward an alternative view: that MSS is partially psychological but is also likely the result of a Bayesian reasoning failure that leads to inflation bias of the posterior probability due to base rate neglect. This Bayesian reasoning failure is a poor probabilistic judgement that occurs as a result of a misunderstanding of the likelihood of an outcome given the symptoms; that either causes or is caused by the psychological stressors that underpin the traditional explanation of MSS. This reflects the focus that medical schools place on students to develop a robust knowledge of pathophysiology, but without a focus on an understanding of statistics and epidemiology - despite both playing a critical role in diagnostic reasoning. Without a cognitive framework for the logical errors that cause MSS, it is not possible to explain why students make these errors in a patterned way, or how the syndrome can be overcome. A theory-informed observational cross-sectional study was performed to explore the prevalence of Bayesian reasoning errors amongst medical students. These results were combined with a theoretical framework to provide a mechanistic explanation of how these Bayesian reasoning errors can cause MSS.

## Methodology

### Study participants

All medical students freely volunteered and gave informed consent to participate in the research. Participants were made aware of their right to not complete, or to withdraw from the study during the anonymised data collection process. Data collection took place throughout the month of November 2024, and all complete responses were included in the final analysis presented in this paper. Anonymity of the participants was maintained throughout.

### Data collection

The survey was advertised to students through posters with a QR code to an online form that was used to collect anonymous responses. The survey was advertised to students in their clinical years who were based in an acute care hospital. Thus, the respondents had completed their preclinical training which includes a module of public health and epidemiology. In order for responses to be collected, the form had to be submitted, which could only be done after answering all sections. This process made sure that only completed responses were recorded which further simplified the data management and analysis process. Researchers have repeatedly failed to substantively prove a link between demographics, academic performance and MSS prevalence or severity [1, 9, 10]. Thus, similar data was not collected or analysed as it was not deemed particularly valuable in evaluating the underlying hypothesis of a generalizable mechanism of MSS. The survey instrument used was developed specifically for this research and a copy is provided in Supplementary Material 1.

### Data analysis

Descriptive and inferential statistics were used to report the results. Where responses involved a written component (respondents were asked to explain why they believe their diagnosis was incorrect) the responses were grouped into broad categories based on the type of error the respondent believed they had made. This was done to enable effective analysis and provide a fair representation of qualitative data. Respondents who entered brief, nonsensical or single character answers were deemed to have done so to enable submission and were categorized as ‘no response’.

### Kahneman and Tversky’s bayesian reasoning question

Kahneman and Tversky (1973) [11] described the widespread issue of Bayesian reasoning errors due to base rate neglect and the representativeness heuristic with their ‘Librarian vs. Farmer’ experiment that asked Americans: “Steve is very shy and withdrawn, invariably helpful but with very little interest in people or in the world of reality. A meek and tidy soul, he has a need for order and structure, and a passion for detail. Is Steve more likely to be a librarian or a farmer?” The majority of respondents believed Steve to be a librarian, despite there being five times as many farmers as librarians in the US at the time. This question was used as a non-medical control question in the study.

### Study design

Participants were asked a series of questions in order to attempt to identify any link between Bayesian error and MSS. The study included the following components:

1. Self-reporting any history of MSS (which has been the standard method of collecting data on MSS prevalence in prior studies as no validated MSS scoring framework exists) [1, 2, 9].
2. A medical version of Kahneman and Tversky’s (1973) [11] ‘librarian or farmer’ question that tested the same principle of base rate neglect. Respondents randomly received one of two questions: one requiring the participant to choose between ischemic stroke and hemiplegic migraine, and another between pheochromocytoma and panic disorder.
3. Kahneman and Tversky’s (1973) ‘librarian or farmer’ question.
4. Participants ranked the following components in order of importance when making a diagnosis: anatomy, physiology, pathophysiology, histology, clinical symptoms of the presenting complaint, other elements of the patient history besides the presenting complaint, clinical signs and physical examination findings, results of other investigations (bloods, imaging, special tests), epidemiology.
5. Students were asked if they had prior knowledge and had been taught about the diseases asked in question 2.
6. The answers were revealed, and a text box was given for the participant to explain why they thought they had gotten the answer wrong, if they had.

The survey was reviewed by a group of four medical students prior to distribution to ensure clarity of the questions. This process also revealed no evidence of excess difficulty or unfamiliarity with the pathologies included. This, combined with the use of a control question on Bayesian reasoning and a ranking of the components used in respondents’ diagnostic reasoning supports the survey’s content validity. As this is the first time the survey instrument was used the construct and convergent validity is shown in the results section.

## Results

At the end of the collection period, all 112 completed responses were included in the data analysis. Table 1 provides a summary of results and shows that over half (63/112) of respondents reported a history of MSS. Both medical questions were answered poorly with less than half of respondents answering correctly (48% and 33%). However, these accuracies are broadly similar to the 41% accuracy when answering Kahneman and Tversky’s (1973) ‘librarian v farmer’ question. There is a significant relationship between respondents with a history of MSS and answering at least one question incorrectly, ${{\upchi}}^{2}\left(1,N=112\right)=37.89,p=0.015)$.

**Table 1:** Summary survey results

Survey component	*n*	Percentage
History of MSS	63	56.25
Answered stroke vs. migraine question	58	51.78
Correctly answered stroke vs. migraine question	28	48.27
Answered pheochromocytoma vs. panic disorder question	54	48.21
Correctly answered pheochromocytoma vs. panic disorder question	18	33.33
Correctly answered librarian vs. farmer question	46	41.07
History of MSS and answered both questions correctly	9	14.29
Had prior knowledge of topics tested	112	100

Furthermore, 47 of the 66 (71%) respondents who answered the medical questions incorrectly also answered Kahneman and Tversky’s question incorrectly (95% CI 59–81%) and were more likely to do so than those that answered the medical questions correctly (RR 1.72, 95% CI 1.18–2.52). Of the 46 of those who answered the medical questions correctly, only 19 (41%) went on to answer Kahneman and Tversky’s question incorrectly (95% CI 28–56%). Given that the expected value (EV) of correct responses to Kahneman and Tversky’s question is 50% with random guessing (as there were only two options), and both groups demonstrate substantive deviations from this (and even larger deviations from each other), the results are strongly suggestive of an underlying process driving discrimination in responses. Only 14% of students with a history of MSS answered both questions correctly (95% CI 6–23%), compared with an EV of 25% with random guessing. There is a significant relationship between performance on the medical questions and performance on the librarian v farmer question, ${{\upchi}}^{2}\left(1,N=112\right)=10.02,p=0.002.$ All respondents reported prior knowledge of the medical pathologies included in the questions they received.

Table 2 shows the mean rank position of each component of diagnostic reasoning, with pathophysiology being the most important component, followed by the symptoms of the presenting complaint. Epidemiology was the lowest ranked component (mean ranking 8.8/9) with most students deeming it the least important factor, yet, as shown in Table 3, when analysing their own errors over half of students who answered a question incorrectly believed their error was related to their consideration of the likelihood of the involved pathologies.

**Table 2:** Importance of components in diagnostic reasoning

Component	Mean Rank Position
Pathophysiology	1.79
Clinical symptoms of the presenting complaint	2.40
Other investigations	3.38
Physiology	3.81
Anatomy	4.80
Physical examination	5.43
Histology	6.79
History other than presenting complaint	7.80
Epidemiology	8.80

**Table 3:** Categorisation of self-analysis of errors in medical questions

Reason	Number of responses	Percentage of completed responses
Other diagnosis more likely	29	50.87
Insufficient knowledge of pathologies tested	15	26.31
“Answered too quickly”	11	19.29
Clicked wrong answer by mistake	2	03.50
No response	9	

## Discussion

### Bayes’ Theorem

Bayes’ Theorem is a normative framework that describes the mathematically optimal estimation of a hypothesis’ probability given new information. It accounts for the prior probability (the base rate of a process), $P\left(A\right)$, the overall probability of observing a particular outcome, $P\left(B\right)$, and the conditional probability of the observed outcome occurring in association with the process in question, $P\left(B\right|A)$.

$$P\left(A|B\right)=\frac{P\left(B|A\right)P\left(A\right)}{P\left(B\right)}$$

This can be used to reflect the necessity of considering the prior probability when making a diagnosis, as an accurate diagnosis is not simply a reflection of the new information given by the patient (their symptoms) but also the underlying prevalence of the diagnosis the physician is considering. The use of an inaccurate prior probability or completely ignoring it altogether is known as base rate neglect. Base rate neglect may occur as a result of the representativeness heuristic – the assessment of a probability based on how well the new information fits a stereotypical occurrence of the disease in question. This manifests as a clinician or student reasoning that a patient’s symptoms alone are sufficient to conclude that a certain diagnosis is likely, even if it is exceedingly rare.

### Fast and slow

Kahneman (2011) [12] described Dual Process Theory (DPT) as a model for human reasoning that considers decisions to be reached via two distinct systems – one fast and one slow. The fast system acts intuitively and makes use of heuristics such as pattern identification. This system is critical for survival but is naturally error-prone given the lack of depth of thought. The second system involves slower, deliberate decision making based on greater analysis and, situation permitting, is used to override or otherwise temper the fast system.

### Integration of study results

**Fig. 2:**
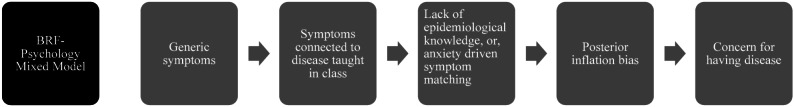
Mixed BRF - Psychology Model for the MSS Process

MSS can be thought of as arising partially from psychological factors (anxiety and hypochondria), and partially as the result of representativeness-triggered base rate neglect. Figure 2 reflects this mixed model and shows how the psychological factors may drive symptom matching behavior that leads to transient inflation of the posterior. This is a failure of moderation by the slow system in DPT. Psychological factors may also play a role in determining who is more prone to MSS as in a class with the same statistical education not everyone develops MSS.

The poor response to Kahneman and Tversky’s original question shows that a substantial number of students exhibit base rate neglect. This is further demonstrated in the poor responses to medical questions testing the same principle, and the overwhelming dismissal of epidemiology as an important factor in diagnostic reasoning. This reflects both an underlying statistical error, but also a possible tendency towards relying heavily on buzzwords or classic presentations in the question stem. While this is a good way to train students to not miss a rare diagnosis, this approach may be doing so at the expense of always correctly making the common or most likely diagnosis. Therefore, while the use of classic presentations may be necessary to test knowledge through a single best answer multiple-choice format; it is not without its shortcomings. Training programs should consider integrating a broad array of assessments to mitigate this by providing more direct testing and feedback of clinical reasoning skills. The risk of letting these reasoning errors go uncorrected is that medical students graduate as doctors that practice with a great appreciation for data interpretation, but a poor gestalt, as one has developed a diagnostic approach high in sensitivity but low in specificity.

One could argue that the results presented here are merely a reflection of stronger students performing better at a challenging task. This interpretation, however, is left wanting when one considers that prior studies show no significant connection between academic performance and MSS prevalence [1, 2, 9].

By discretising the respondent’s self-analyses of error, it is clear that applying Bayesian reasoning in clinical decision making is a systemic issue. In this study, nearly three quarters of students did not believe their knowledge of the subject matter was the major cause of their error. Additionally, the 29 of 57 respondents that identified another diagnosis being more likely as the cause of their error may be an underrepresentation of the scale at which posterior inflation bias is occurring. This is because it is possible (and likely) that there are students that do not truly realise that they are making this error. This has been reflected in other research, including Kahneman and Tversky’s (1973) [11] work, where many participants refused to admit error in the ‘Linda problem,’ where respondents believed that the occurrence of a sequence of events were more probable than a single event.

Unfortunately, the popularity of the psychological explanation for MSS has led to the wrong questions being posed and answered by researchers. Instead of seeking to better understand the cognitive error that leads to MSS studies have been largely focused on manifestations of the syndrome. This area of the literature is hotly contested - with multiple studies that both support and fail to support the existence of MSS based on manifestations of it, such as anxiety, seeking healthcare, or lower GPAs [1, 9]. Describing MSS purely as a type of hypochondriac behaviour also fails on one critical point: it does not lead to an increase in physician consultations as true hypochondria would [9]. Whilst some argue that this indicates MSS is not real (which stands against the litany of recorded and experienced evidence), it demonstrates a critical flaw with researchers trying to shoehorn MSS into psychological pathologies without considering alternative contributing factors.

### Solutions

Bayesian reasoning is teachable and easily applied to diagnostic reasoning [13]. If medical students are intuitively matching their symptoms to diseases based on prototype fit, then they must be trained and examined in a manner that moves beyond this. A more comprehensive statistical curriculum with a focus on reasoning and the integration of epidemiology in decision making would be well-suited to solving this issue and would also address the lack of confidence students have with applying statistics to research. Oral long cases and essay-based examinations offer examiners the opportunity to interrogate a student’s clinical reasoning. Evaluating students in this manner may test and stress the importance of methodical clinical reasoning. However, these suggestions are hypothesis-generated and not proven by the findings of this paper.

### Limitations

Information on symptom duration was not collected which may aid in ruling out genuine incipient illnesses. However, this is unlikely to meaningfully change the results given the rarity of such conditions in the sample studied. The study’s use of self-recruitment induces selection bias which may artificially alter the prevalence of MSS and reasoning errors in the sample. The study was conducted at a single institution and does not include students from all academic years, which reduces generalizability. The survey’s binary answer choices and forced ranking of diagnostic components reduces the value of and ability to perform prior statistical validity testing. No demographic information was collected. The QR-code-based responses, and the anonymity of these responses, mean that no external validation of student responses could occur.

### Future research

This paper suggests Bayesian reasoning failures contribute to MSS and thus replication studies at other institutions using multiple years of students would be valuable in determining the generalizability of the conclusions drawn. Further development of the survey instrument is necessary to increase the variety of possible questions a participant may receive and enable analysis of the relationship between Bayesian and clinical reasoning abilities. Survey development should also ensure question representativeness and standardization of difficulty. This would decrease the risk of bias and support the effort to draw broadly applicable conclusions. Further studies investigating the role of psychological factors in MSS would be valuable in expanding and updating the current evidence base.

## Conclusion

This paper used a testable experimental approach to provide a new mechanistic account of a plausible cognitive pathway of MSS. In this mixed model MSS likely reflects a consistent but correctable deviation from Bayesian reasoning combined with psychological factors. This may stem from an overreliance on pattern recognition (the representativeness heuristic), as well as inflation bias of the posterior probability due to either a poor knowledge, or the total exclusion of epidemiology in the student’s diagnostic calculus. Students responded poorly to both a classic question testing Bayesian reasoning, as well as medical equivalents. Over half of those who made an error on medical questions did so because they believed another diagnosis was more likely, reflecting an inaccurate knowledge of prior probabilities. Improvement in the statistics curriculum for medical students and avoiding an overreliance on multiple-choice examinations were presented as plausible avenues to diversify the type of reasoning and information medical students must apply. Together, these offer the student a new approach to problem solving that will hopefully not just reduce the incidence of MSS but increase depth of thought in the clinical environment and positively affect patient care.

## Supplementary Information

Supplementary Material 1.

## Data Availability

The dataset generated by this study is not publicly available, but summary anonymised data may be available on reasonable request.
