# Supplementary material for: Medical student syndrome: a bayesian reasoning failure
Source: BMC Med Educ. 2026 Mar 16;26:659. doi: 10.1186/s12909-026-09018-9 (PMC13104304; doi:10.1186/s12909-026-09018-9)
Supplement: Supplementary file 1 — Supplementary Material 1. [file 12909_2026_9018_MOESM1_ESM.docx]

**Supplementary Material 1**

**English Copy of Survey Instrument**

**Title: Medical Student Syndrome and Diagnostic Reasoning Survey**

**Instructions to participants**

This survey explores diagnostic reasoning among medical students. Participation is voluntary and all data collected is anonymous. You may stop at any time prior to submission. All responses are anonymous and cannot be linked back to you. Please answer all questions honestly and to the best of your ability.

**Section 1: Medical Student Syndrome**

1. Have you ever experienced Medical Student Syndrome, defined as becoming concerned that you may have a serious or rare illness based on symptoms you experience during your medical training and relate to a disease or pathology you have learned about?

☐ Yes

☐ No

**Section 2: Medical Diagnostic Reasoning**

Participants were randomly assigned to receive one of the following two questions.

1. A. A patient presents with acute onset unilateral weakness and sensory disturbance. They report headache and visual symptoms. Neurological examination is otherwise unremarkable, and symptoms resolve completely within several hours. Which diagnosis is more likely?

☐ Ischaemic stroke

☐ Hemiplegic migraine

1. A patient presents with episodic palpitations, headache, sweating and anxiety. Blood pressure is elevated during episodes but is otherwise normal. Which diagnosis is more likely?

☐ Pheochromocytoma

☐ Panic disorder

**Section 3: Bayesian Reasoning Task**

1. Consider the following description: Steve is very shy and withdrawn, invariably helpful but with very little interest in people or in the world of reality. A meek and tidy soul, he has a need for order and structure, and a passion for detail. Which is more likely?

☐ Steve is a librarian

☐ Steve is a farmer

**Section 4: Diagnostic Reasoning Prioritisation**

1. When making a diagnosis, please rank the following components in order of importance (1= most important, 9 = least important).

☐ Clinical symptoms of the presenting complaint

☐ Other elements of the patient history (besides the presenting complaint)

☐ Anatomy

☐ Physiology

☐ Epidemiology

☐ Results of investigations (blood tests, imaging, special tests)

☐ Pathophysiology

☐ Clinical signs and physical examination findings

☐ Histology

**Section 5: Prior Knowledge**

1. Prior to this survey, had you been taught about or were you familiar with the medical conditions presented in the diagnostic question you received?

**Section 6: Reflection on Diagnostic Reasoning**

The correct answers to the diagnostic reasoning questions were revealed.

1. If you answered the medical diagnostic question incorrectly, please briefly explain why you believe you selected the incorrect answer.

(Open text response)

**End**
